# Clinical features and outcomes of hypocellular acute myeloid leukemia in adults: A Korean AML registry data

**DOI:** 10.1097/MD.0000000000024185

**Published:** 2021-01-08

**Authors:** Ik-Chan Song, Deog-Yeon Jo, Hyeoung-Joon Kim, Yoo-Hong Min, Dae Sik Hong, Won-Sik Lee, Ho-Jin Shin, Je-Hwan Lee, Jinny Park, Hee-Je Kim

**Affiliations:** a Department of Internal Medicine, Chungnam National University Hospital, Daejeon; b Division of Hematology-Oncology, Chonnam National University Hwasun Hospital, Hwasun, Jeollanam-do; c Department of Internal Medicine, Yonsei University College of Medicine, Seoul; d Department of Hemato-Oncolgy, Soon Chun Hyang University Hospital, Bucheon; e Department of Internal Medicine, Inje University Busan Paik Hospital, Busan; f Division of Hematology-Oncology, Department of Internal Medicine, School of Medicine, Pusan National University Hospital, Pusan; g Department of Hematology, Asan Medical Center, University of Ulsan College of Medicine, Seoul; h Division of Hematology-Oncology, Department of Internal Medicine, Gachon University Gil Hospital, Incheon; i Department of Hematology, Leukemia Research Institute, Catholic Hematology Hospital, Seoul St. Mary's Hospital, College of Medicine, The Catholic University of Korea, South Korea.

**Keywords:** acute myeloid leukemia, hypocellular, outcome, survival

## Abstract

The hypocellular variant of acute myeloid leukemia (AML) is defined as bone marrow cellularity of <20% in a biopsy specimen at presentation. We performed a retrospective analysis of the clinical features and survival outcomes of hypocellular AML in a Korean population. We reviewed the medical records of all patients diagnosed with AML at nine hospitals participating in the Korean AML registry from 2006 to 2012. Overall survival (OS) and event-free survival (EFS) rates were calculated from the time of diagnosis until death or an event, respectively. In total, 2110 patients were enrolled and 102 (4.8%) were identified as having hypocellular AML. Patients with hypocellular AML were older than those with non-hypocellular AML (median age: 59 vs 49 years; *P* < .001) and presented with leukopenia more frequently (mean white blood cell count: 5810/μL vs 40549/μL; *P* < .001). There was no difference between patients with and without hypocellular AML in terms of the presence of antecedent hematologic disorders (5.9% vs 5.3%; *P* *=* .809). *FLT3*-ITD and *NPM1* mutations were less common in hypocellular than non-hypocellular AML (*FLT3*-ITD mutations: 1.2% vs 14.3%, *P* < .001; *NPM1* mutations: 0% vs 9.5%, *P* = .019). No differences were seen between the hypocellular and non-hypocellular AML groups in the complete remission rate (53.9% vs 61.3%, *P* = .139) or early death rate (defined as any death before 8 weeks; 14.7% vs 13.0%, *P* = .629). The OS and EFS did not differ between the hypocellular and non-hypocellular AML groups (median OS: 16 vs 23 months, *P* = .169; median EFS: 6 vs 9 months, *P* = .215). Hypocellular AML is more frequently observed in older-aged patients and have fewer *FLT3*-ITD and *NPM1* mutation, but the clinical outcomes of hypocellular AML do not differ from those of non-hypocellular AML.

## 1 Introduction

Acute myeloid leukemia (AML) is a heterogeneous group of hematopoietic cell malignancies originating from myeloid precursor cells and varying in pathogenesis.^[1,2]^ The cellularity of bone marrow in patients with AML is usually above average due to the presence of malignant cells.^[3,4]^ However, a small number of patients are unusual in having cellularity below the average for their age.^[5–7]^ Diagnosis and treatment of hypocellular AML patients is challenging for hematologists because of overlap in the features of hypocellular myelodysplastic syndrome and aplastic anemia, including with respect to the potential for prolonged cytopenia and early mortality during chemotherapy.^[8,9]^

A recently published study conducted by Al-kali et al at MD Anderson Cancer Center reported that ∼10% of AML patients had hypocellular AML, defined as bone marrow cellularity of <20% in a biopsy specimen at presentation.^[10]^ Patients with hypocellular AML were older than those with non-hypocellular AML, and presented with cytopenias more frequently. Al-kali et al also reported that the treatment outcomes and prognosis of hypocellular AML did not differ from those of non-hypocellular AML. However, most studies have been based on data from a small number of patients or a single institute. Therefore, we used Korean AML/MDS Working Party registry data to conduct a nationwide population study of the clinical features, molecular features, and prognosis of hypocellular AML.

## 2 Materials and methods

### 2.1 Patients and baseline evaluation

We conducted a retrospective study of all patients newly diagnosed with AML at nine hospitals participating in the Korean AML registry between 2006 and 2012. The data cut-off point was October 2014. For all patients, core biopsies and bone marrow aspirate smears were completed at the time of presentation. Bone marrow aspirate smears were assessed using Wright-Giemsa stain followed by cytochemical analysis for myeloperoxidase and non-specific esterase. Cytogenetic analysis was performed using G-banding, with at least 20 metaphases counted. *FMS*-like tyrosine kinase 3-internal tandem duplication (*FLT3*-ITD) mutations and nucleophosmin-1 (*NPM1*) mutations were assessed using genomic DNA extracted from bone marrow aspirates with polymerase chain reaction assays.

Hypocellular AML is defined as AML with a bone marrow cellularity of <20%. We classified the patients according to French-American-British (FAB) and cytogenetic and molecular risk status stratified by National Comprehensive Cancer Network (NCCN) guidelines.

Treatment response was assessed according to criteria proposed by the 2003 International Working Group. For instance, complete remission was defined as the presence of <5% blasts in bone marrow with recovery of the peripheral blood count. Information on antecedent hematologic disorders and prior chemotherapy or radiotherapy was collected.

The study protocol was approved by the Institutional Review Board of Chungnam National University Hospital (Daejeon, South Korea). The requirement for informed consent was waived because of the retrospective nature of the analysis.

### 2.2 Endpoints

The primary endpoints were clinical features, including differences in antecedent hematologic disease, cytopenias, cytogenetics, molecular genetics, complete remission rate, and early mortality. The secondary endpoints were survival outcomes, such as overall survival (OS) and event-free survival (EFS), and neutrophil and platelet recovery time after induction chemotherapy. Early mortality was defined as death from any cause within 2 months after diagnosis. The OS was calculated from the time of diagnosis until death, and EFS from the time of complete response to relapse or death from any cause. Neutrophil recovery was defined as a neutrophil count ≥0.5 × 10^9^/L for 3 days or a neutrophil count ≥1.0 × 10^9^/L for 1 days after the start of induction chemotherapy. Platelet recovery was defined as a platelet count ≥20 × 10^9^/L without a transfusion.

### 2.3 Statistics

Categorical variables were compared using Student's *t* test and the chi-squared test. Logistic regression was used to examine correlations. Overall and EFS were assessed using the Kaplan–Meier method. Survival rates were compared using the log-rank test. A *P*-value < .05 was considered significant. All statistical analyses were performed using SPSS software (ver. 24.0; IBM Corporation, Armonk, NY).

## 3 Results

### 3.1 Patients’ characteristics

A total of 2110 patients were enrolled from nine hospitals participating in the Korean AML registry (based on WHO criteria) between January 2006 and December 2012. Of these patients, 102 (4.8%) had a bone marrow cellularity of <20% and were diagnosed with hypocellular AML. The characteristics of patients with hypocellular AML and non-hypocellular AML are presented in Table 1. The median age of patients with hypocellular AML was 59 years (range: 18–88 years). They were significantly older than patients with non-hypocellular AML (*P* < .001). The hypocellular AML group had a median bone marrow cellularity of 15% (range: 5–20%). The gender ratio and rate of antecedent hematologic disease did not differ by group. The median white blood cell count was 1690/uL (range: 470–239,620/uL) in the hypocellular AML group, which was significantly lower than that in the non-hypocellular AML group (*P* < .001). The most common subtype of AML according to the FAB classification was M2. This subtype accounted for 38.2% and 36.0% of hypocellular and non-hypocellular AML cases, respectively. However, the unclassifiable subtype was statistically significantly higher in the hypocellular AML group than in the non-hypocellular AML group (*P* < .001).

**Table 1:** Baseline characteristics of patients (N = 2110).

Parameters	h-AML (n = 102)	Non h-AML (n = 2008)	*P*
Age (yr), median (range)	59.0 (18–88)	49.0 (18–95)	<.001^∗^
Gender, M: F (%)	65:37 (63.7:36.3)	1081:927 (53.8:46.2)	.050^§^
Antecedent hematologic disorder, N (%)	6 (5.9)	107 (5.3)	.166^∗^
BM cellularity (%), median (range)	15.0 (5–20)	90.0 (25–100)	<.001^∗^
WBC (/μL), median (range)	1690 (470–239,620)	11,635 (290–484,090)	<.001^∗^
Platelet (×10^3^/μL), median (range)	63 (8--423)	49 (3–481)	.831^∗^
Hb (g/dL), median (range)	8.3 (2.8–13.4)	8.4 (1.7–13.0)	.693^∗^
FAB classification, N (%)			
M0	14 (13.7)	71 (3.5)	
M1	9 (8.8)	319 (15.9)	
M2	39 (38.2)	723 (36.0)	
M3	6 (5.9)	212 (10.6)	
M4	7 (6.9)	304 (15.1)	<.001^§^
M5	2 (2.0)	106 (5.3)	
M6	3 (2.9)	57 (2.8)	
M7	1 (1.0)	29 (1.4)	
Unclassifiable	21 (20.6)	187 (9.2)	

### 3.2 Cytogenetic and molecular genetics

Risk status based on validated cytogenetics according to NCCN guidelines did not differ between the two groups (Table 2). Intermediate risk status was the most common status in both groups (62.7% of hypocellular AML patients and 53.4% of non-hypocellular AML patients, *P* = .104). Rates of chromosomal abnormalities that are frequently observed in MDS, such as deletion 5 or 5q and deletion 7 or 7q, were also similar (−5 or −5q: 4.9% of hypocellular AML patients and 3.9% of non-hypocellular AML patients, *P* = .626; −7 or −7q: 8.8% of hypocellular AML patients and 5.0% of non-hypocellular AML patients, *P* = .087). Rates of translocation *t*(8;21) and of chromosome 16 inversion or translocation *t*(16;16) were also similar in both groups (*t*(8:21): 8.8% of hypocellular AML patients and 8.7% of non-hypocellular AML patients, *P* = .956; inv(16) or *t*(16:16): 1.0% of hypocellular AML patients and 3.8% of non-hypocellular AML patients, *P* = .136). A total of 1778 patients (64.3%) were tested for the *FLT3*-ITD mutation, and 883 patients (41.8%) were tested for the *NPM1* mutation. None of the hypocellular AML patients tested had the *NPM1* mutation, compared to 79 of the 830 non-hypocellular patients tested (9.5%) (*P* = .019). The *FLT3*-ITD mutation was found in 1 of the 84 hypocellular AML patients tested (1.2%) and in 242 of the 1694 non-hypocellular AML patients tested (14.3%) (*P* < .001).

**Table 2:** Cytogenetic and molecular characteristics at diagnosis (N = 2,110).

	h-AML (N = 102)	Non h-AML (N = 2008)	*P*^§^
Cytogenetic risk stratification			.104
Favorable	16 (15.7%)	442 (22.0%)	
Intermediate	64 (62.7%)	1073 (53.4%)	
Poor	18 (17.6%)	460 (22.9%)	
Not evaluated	4 (3.9%)	33 (1.7%)	
–5 or –5q	5 (4.9%)	79 (3.9%)	.626
–7 or –7q	9 (8.8%)	100 (5.0%)	.087
Translocation (8;21)	9 (8.8%)	174 (8.7%)	.956
Inversion (16) or *t*(16:16)	1 (1.0%)	77 (3.8%)	.136
*NPM1* mutation	0/53 (0.0%)	79/830 (9.5%)	.019
*FLT3*-ITD mutation	1/84 (1.2%)	242/1694 (14.3%)	<.001

### 3.3 Clinical outcomes

In total, 64 of 102 patients in the hypocellular AML group (63.7%) and 1480 of 2008 patients in the non-hypocellular AML group (73.7%) were treated with standard induction chemotherapy. This difference was not statistically significant (*P* = .112). Idarubicin plus cytarabine or enocitabine was most common initial induction regimen in both groups, followed by daunorubicin plus cytarabine or enocitabine (Table 3). In total, 28 patients with hypocellular AML (27.5%) and 866 with non-hypocellular AML (43.1%) underwent allogenic hematopoietic stem cell transplantation (*P* = .002).

**Table 3:** Induction chemotherapy and allogeneic hematopoietic stem cell transplantation (N = 2110).

	h-AML	Non h-AML	
	(N = 102)	(N = 2008)	*P*^§^
Induction therapy, N (%)			
IDA + (ARA or BHAC)	55 (53.9)	1,259 (62.7)	.112
DNRV + (ARA or BHAC)	9 (8.8)	221 (11.0)	
Others	23 (22.5)	325 (16.2)	
Supportive care only	15 (14.7)	203 (10.1)	
Allo-HSCT, N (%)	28 (27.5)	866 (43.1)	.002

**Figure 1:**
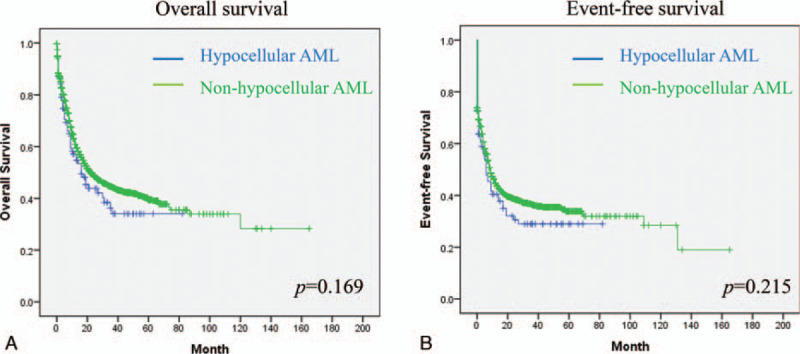
(A) Overall survival and (B) event-free survival between hypocellular AML and non-hypocellular AML (N = 2110).

The complete remission rate after induction chemotherapy was 53.9% in the hypocellular AML group and 61.3% in the non-hypocellular AML group (*P* = .139). The early mortality rate was 14.7% in the hypocellular AML group, which was similar to the rate in the non-hypocellular AML group (13.1%, *P* = .629, Table 4). The median OS in the hypocellular AML group was 16 months (range: 8.9–23.0 months), which was not significantly different from the median OS in the non-hypocellular AML group (23.0 months; range: 19.2–26.8, *P* = .169, Fig. 1A). The median EFS was 6 months (range: 2.6–9.4 months) in the hypocellular AML group, which was not significantly different from the median EFS in the non-hypocellular AML group (9.0 months; range: 7.8–10.2 months, *P* = .215, Fig. 1B).

**Table 4:** Clinical outcomes (N = 2110).

Outcomes	h-AML (N = 102)	Non h-AML (N = 2008)	*P*
CR, N (%)	55 (53.9)	1,230 (61.3)	.139^§^
Early death, N (%)	15 (14.7)	227 (13.1)	.629^§^
Overall survival (mo), median (95% CI)	16.0 (8.9–23.0)	23.0 (19.2–26.8)	.169^∗^
Event-free survival (mo), median (95% CI)	6.0 (2.6–9.4)	9.0 (7.8–10.2)	.215^∗^

An analysis of bone marrow recovery after induction chemotherapy in patients from a single institute (N = 348) showed no difference in the median neutrophil recovery time between the hypocellular AML group (21.5 days; range: 16–56 days) and the non-hypocellular AML group (median, 21.0 days; range: 13–77 days) (*P* = .925). The median platelet recovery time also was similar, at 22.5 days (range: 15–57 days) in the hypocellular AML group and 22.0 days (range: 8–58 days) in the non-hypocellular AML group (*P* = .787). However, hypocellular AML patients had lower median cellularity after induction chemotherapy (20%; range: 5–20%) than non-hypocellular AML patients (50%; range: 5–95%) (*P* < .001, Table 5).

**Table 5:** Bone marrow recovery after induction chemotherapy (N = 348).

Outcomes	h-AML (N = 36)	Non h-AML (N = 312)	*P*^§^
ANC recovery (days), median (range)	21.5 (16–56)	21.0 (13–77)	.925
Platelet recovery (days), median (range)	22.5 (15–57)	22.0 (8–58)	.787
BM cellularity (%), median (range)	20.0 (5–80)	50.0 (5–95)	<.001

## 4 Discussion

In this registry-based data analysis, hypocellular AML was seen more frequently in older patients, who also tended to present with more profound cytopenias. Hypocellular AML did not differ from non-hypocellular AML in terms of cytogenetic character, treatment response, or survival outcomes. However, *FLT3*-ITD and *NPM1* mutations were less common in hypocellular AML than in non-hypocellular AML.

Hypocellular AML was seen in 4.8% of 2110 AML patients. In previously published reports, its frequency ranged from 5% to 10% among all cases of AML.^[10,11]^ In a retrospective analysis of patients at MD Anderson Cancer Center, Al-Kali et al reported that about 9% of 1342 patients had hypocellular AML.^[10]^ In a study of cases at a single Chinese center, Hu et al found that 7.4% of 631 patients were diagnosed with hypocellular AML.^[11]^ The marrow hypoplasia present in this subtype of AML makes it more difficult to distinguish from hypocellular myelodysplastic syndrome or aplastic anemia. In one study, the concordance rate for the diagnosis of hypocellular AML in a group of hematopathologists was only 57%.^[8]^ Despite these diagnostic challenges, the frequency of hypocellular AML appears to be relatively lower in the eastern population than in the western. The Korean AML registry that used in this study holds data from major centers in Korea that treated patients with AML, so this data represents the frequency of hypocellular AML in Koreans.

In this study, hypocellular AML was observed in older patients and associated with more severe leukopenia. This is consistent with the findings of previous studies.^[10,11]^ Al-kali et al reported that hypocellular AML was characterized by older age (median age, 65 years) and profound cytopenias.^[10]^ Also, Hu et al found that hypocellular AML was associated with lower white blood cell counts and older age (median age, 56 years).^[11]^ In both studies, hypocellular AML was also more frequently associated with antecedent hematologic disorders. This trend was not replicated in our study, which is thought to be the result of the limited amount of past medical history data in registry-based studies. To compensate for this limitation, we analyzed chromosomal abnormalities frequently observed in MDS. Rates of chromosomal abnormalities (specifically, deletions in chromosomes 5 and 7) did not differ between the hypocellular and non-hypocellular AML groups. Al-kali et al reported similar findings in the chromosomal abnormalities.^[10]^

The *FLT3*-ITD mutation is found in about 20% to 30% of de novo AML patients.^[12,13]^ In hypocellular AML, we found a markedly lower rate of *FLT3*-ITD. This result is consistent with the studies by Al-kali et al and Hu et al. The *FLT3*-ITD mutation is a well-known adverse prognostic factor in AML and the patients with the *FLT3*-ITD mutation had increased white blood cell counts, because it suppresses apoptosis and proliferates the dysregulated cells.^[14–16]^ Despite the low frequency of the *FLT3*-ITD mutation in hypocellular AML patients, the prognosis is similar to that of non-hypocellular AML patients. It is thought that the poor prognosis caused by *FLT3*-ITD mutation was offset in non-hypocellular AML patients, because those were relatively younger and had undergone more allogeneic hematopoietic stem cell transplants than hypocellular AML patients. The *NPM1* mutation was also positively correlated with high white blood cell counts in our AML patients, and it frequently found in conjunction with the *FLT3*-ITD mutation.^[17,18]^ The *NPM1* mutation is present in ∼30% of AML cases, but it is less common in hypocellular AML than in non-hypocellular AML. *NPM1* mutation is favorable prognostic factor in AML patients in the absence of *FLT3*-ITD mutation.^[19,20]^ However, hypocellular AML patients had significantly lower rate of both *NPM1* mutation and *FLT3*-ITD mutation in this study. Therefore, it is thought that the prognostic effect of the *NPM1* mutation was weakened in hypocellular AML patients.

Hypocellular AML was expected to be associated with a higher mortality rate or a slower rate of bone marrow recovery after induction chemotherapy, due to its profound cytopenias, but early mortality (death before 8 weeks) and bone marrow recovery time did not differ between the hypocellular and non-hypocellular AML groups. Interestingly, the hypocellular AML cases retained low cellularity after BM recovery of induction chemotherapy, which data has not been previously reported in other articles. This suggests that patients with hypocellular AML are unique in having low BM cellularity regardless of leukemia. The development and increasingly widespread use of molecular genetic technologies, such as next-generation sequencing and single-cell RNA sequencing, is expected to shed further light on this issue.^[21,22]^ Chen et al, for instance, used single-cell targeted sequencing of leukemic stem cells to demonstrate a nonlinear pattern of clonal evolution from MDS to AML.^[23]^ In our study, non-hypocellular AML patients had a higher rate of allogeneic hematopoietic stem cell transplantation compared to the hypocellular AML patients, but survival outcomes were similar in both groups. This is probably because non-hypocellular AML tended to occur at a younger age than hypocellular AML, but genetic mutations associated with poor prognosis, such as FLT3-ITD, were more frequently observed in non-hypocellular AML.

In conclusion, we found that hypocellular AML was more frequently observed in older patients, who were less likely to exhibit *FLT3*-ITD and *NPM1* mutations. However, the clinical outcomes did not differ between hypocellular and non-hypocellular AML.

## Acknowledgments

We would like to thank all centers of the Korean Society of Hematology AML/MDS Working Party for their contributions to this analysis.

## Author contributions

**Conceptualization:** Ik-Chan Song, Deog-Yeon Jo, Hee-Je Kim.

**Data curation:** Ik-Chan Song, Deog-Yeon Jo, Hyeoung-Joon Kim, Hee-Je Kim.

**Formal analysis:** Ik-Chan Song, Yoo-Hong Min, Hee-Je Kim.

**Funding acquisition:** Ik-Chan Song.

**Investigation:** Ik-Chan Song, Dae Sik Hong, Won-Sik Lee, Ho-Jin Shin, Je-Hwan Lee, Jinny Park, Hee-Je Kim.

**Methodology:** Ik-Chan Song, Hee-Je Kim.

**Project administration:** Ik-Chan Song, Hee-Je Kim.

**Supervision:** Hee-Je Kim.

**Writing – original draft:** Ik-Chan Song, Deog-Yeon Jo.

**Writing – review & editing:** Ik-Chan Song, Je-Hwan Lee, Hee-Je Kim.
